# Endoscopic intermuscular dissection: insights from China on minimally invasive treatment for early rectal cancer

**DOI:** 10.1055/a-2443-3995

**Published:** 2024-11-13

**Authors:** Dejun Fan, Tao Yang, Jingwen Qi, Qiuning Wu, Xutao Lin, Chujun Li, Xianhe Kong

**Affiliations:** 1 Department of Gastrointestinal Endoscopy, The Sixth Affiliated Hospital, Sun Yat-sen University, Guangzhou, China; 2 Guangdong Provincial Key Laboratory of Colorectal and Pelvic Floor Diseases, The Sixth Affiliated Hospital, Sun Yat-sen University, Guangzhou, China; 3 Biomedical Innovation Center, The Sixth Affiliated Hospital, Sun Yat-sen University, Guangzhou, China; 4 Department of Pathology, The Sixth Affiliated Hospital, Sun Yat-sen University, Guangzhou, China

A 73-year-old woman presented with a history of increased bowel movements and intermittent rectal bleeding for 1 month. A colonoscopy revealed tumors in the rectum, which were confirmed through biopsy as adenoma and intraepithelial neoplasia. Staging with computed tomography and magnetic resonance imaging indicated T2N0–1aM0 disease. Endoscopic intermuscular dissection (EID) was subsequently performed ( Video 1 ).

Endoscopic intermuscular dissection is performed for an early rectal cancer, allowing the preservation of the external longitudinal muscle layer.Video 1

The EID procedure began with lesion identification using white-light imaging and chromoendoscopy ( Fig. 1 ), followed by circumferential electrosurgical marking ( Fig. 2 ). A sodium hyaluronate solution mixed with indigo carmine was injected around the lesion. A mucosal incision exposed the submucosal layer, and any superficial bleeding was managed as necessary. Submucosal dissection deepened to the muscularis propria layer, transitioning to intermuscular dissection between the inner circular and outer longitudinal muscular layers ( Fig. 3 ). The lesion was completely resected, and hemostasis was achieved with high-frequency hemostatic forceps. Wound closure was performed as needed ( Fig. 4 ) 1 . Postoperative pathological examination revealed a pT1b tumor with negative vertical margins. The deepest tumor infiltration was 1.1 mm from the basal resection margins ( Fig. 5 ). The patient recovered without complications and was discharged.

**Fig. 1:**
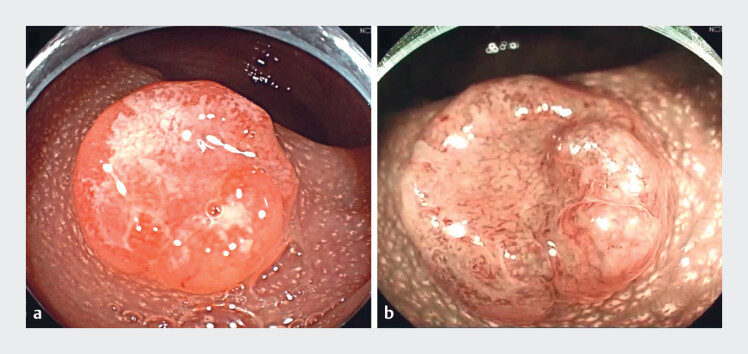
Imaging studies on the tumor. **a** White-light image. **b** Blue-light image.

**Fig. 2:**
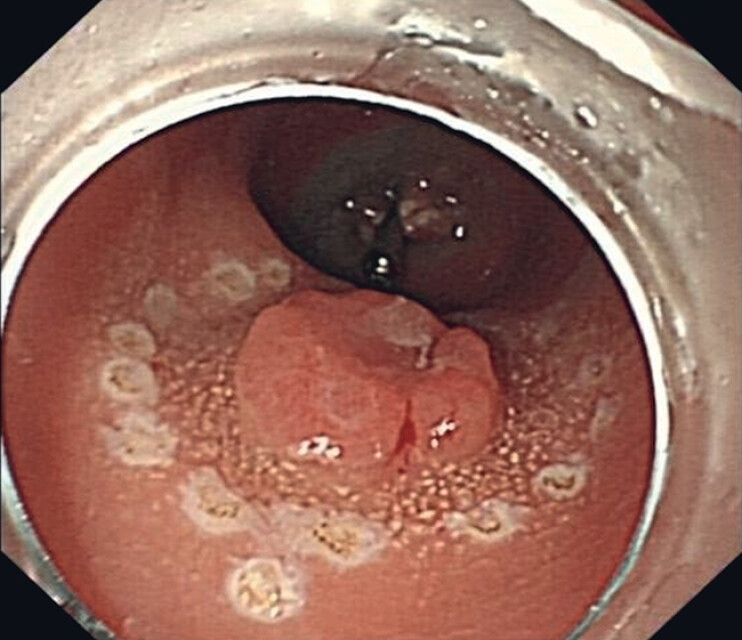
Marking the boundaries of the tumor.

**Fig. 3:**
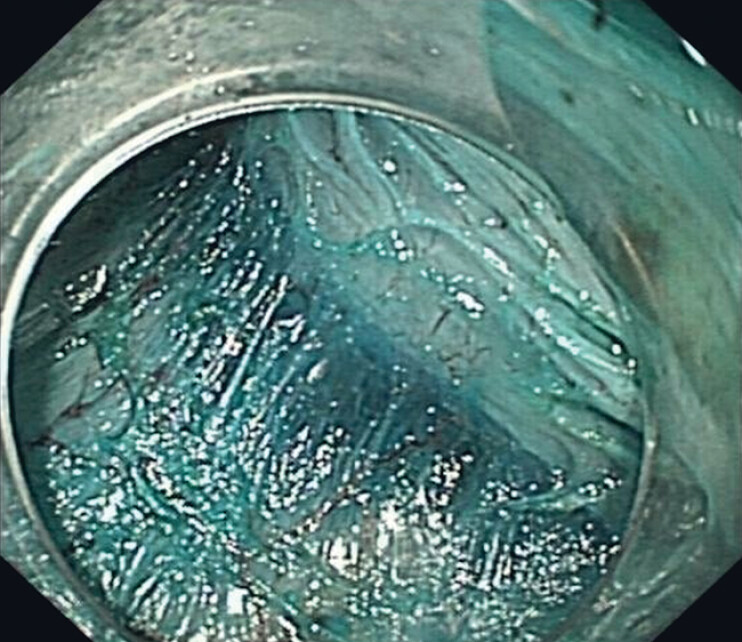
Intermuscular dissection: inner circular muscle excision, outer longitudinal muscle preservation.

**Fig. 4:**
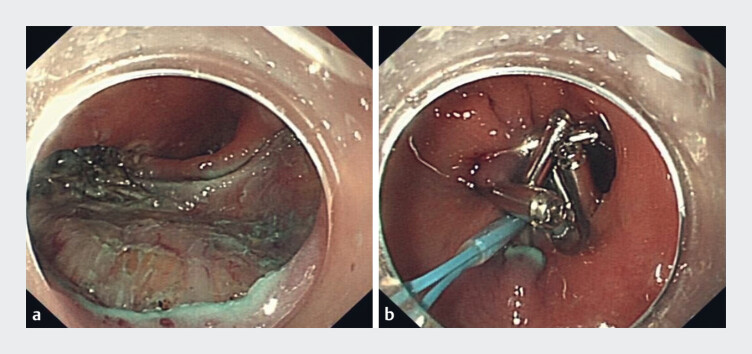
Complete resection of the lesion and complete closure of the wound.

**Fig. 5:**
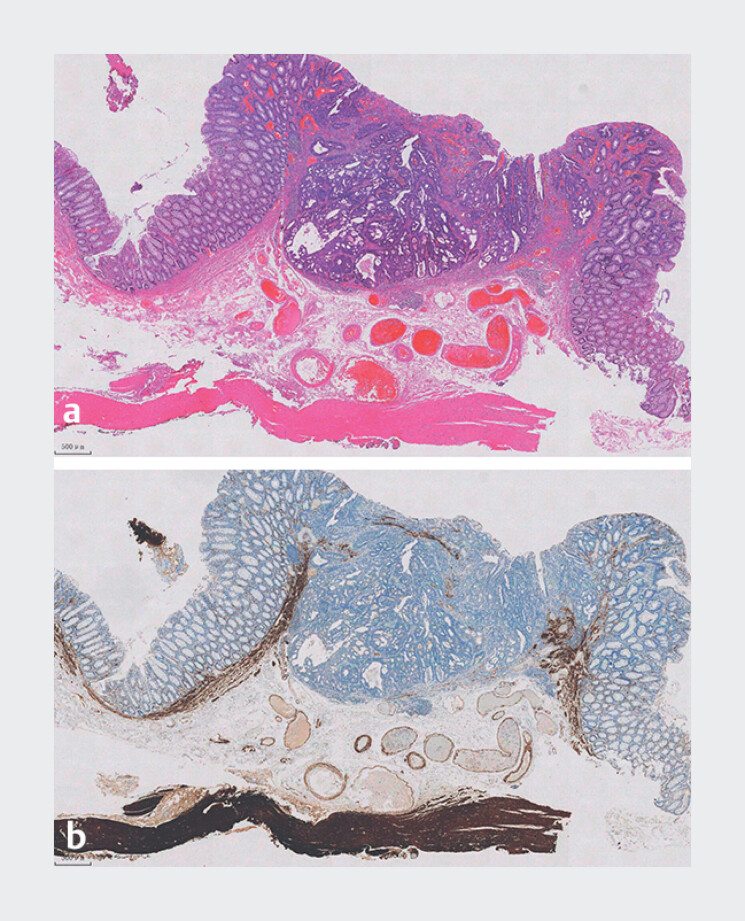
Pathological examination. **a** Hematoxylin and eosin staining. **b** Desmin staining.

EID is highlighted as a promising technique for early rectal cancer with deeper submucosal invasion 2 . The immediate postoperative results show the potential for precise and effective resection. However, the long-term oncological outcomes require further clinical validation. This case demonstrates the technical feasibility of EID in achieving R0 resection with clear margins in a patient with initially suspected T2 rectal cancer, confirmed as T1 postoperatively. Further studies are necessary to establish the long-term efficacy of EID compared with conventional laparoscopic surgery.

Endoscopy_UCTN_Code_TTT_1AQ_2AD_3AD

## Notes

Endoscopy E-Videos https://eref.thieme.de/e-videos
*E-Videos* is an open access online section of the journal *Endoscopy* , reporting on interesting cases and new techniques in gastroenterological endoscopy. All papers include a high-quality video and are published with a Creative Commons CC-BY license. Endoscopy E-Videos qualify for HINARI discounts and waivers and eligibility is automatically checked during the submission process. We grant 100% waivers to articles whose corresponding authors are based in Group A countries and 50% waivers to those who are based in Group B countries as classified by Research4Life (see: https://www.research4life.org/access/eligibility/ ). This section has its own submission website at https://mc.manuscriptcentral.com/e-videos .
